# An unusual case of congenital cytomegalovirus infection-related retinopathy

**DOI:** 10.1186/s12886-016-0246-9

**Published:** 2016-06-07

**Authors:** Mizuki Tagami, Shigeru Honda, Ichiro Morioka, Kazumoto Iijima, Hideto Yamada, Makoto Nakamura

**Affiliations:** Department of Surgery, Division of Ophthalmology, Kobe University Graduate School of Medicine, 7-5-2 Kusunoki-cho, Chuo-ku Kobe, 650-0017 Japan; Department of Pediatrics, Kobe University Graduate School of Medicine, Kobe, Japan; Department of Obstetrics and Gynecology, Kobe University Graduate School of Medicine, Kobe, Japan

**Keywords:** Congenital cytomegalovirus, Neonate, Retinal arteriovenous anastomosis

## Abstract

**Background:**

Cytomegalovirus (CMV) is the most common congenital infection, and chorioretinitis is the most common ophthalmic manifestation of congenital CMV infection. We experienced a unique case of CMV retinopathy showing unusual retinal vessels.

**Case presentation:**

An infant boy weighing 1860 g was born at 36 weeks. He was diagnosed with severe symptomatic congenital CMV infection, which was confirmed by positive CMV-DNA in urine and whole blood, and he was referred to our ophthalmology department on his first day of life. Ophthalmoscopic examination and fluorescein angiography revealed no chorioretinitis but major retinal vascular occlusions and arterio-venous anastomosis associated with CMV detection in the aqueous humor. These findings regressed within a week after treatment with systemic gancyclovir administration.

**Conclusions:**

To our knowledge, there are no reports of these ocular issues associated with congenital CMV infection. These findings may be useful for the early and rapid diagnosis of congenital CMV infection.

## Background

Cytomegalovirus (CMV) is the most common cause of congenital infection throughout the world. The reported prevalence of congenital CMV infection ranges from approximately 0.6 % to 6.1 % of all live births [1]. Of those born with a congenital CMV infection, 10 % are symptomatic at birth, and most commonly present with petechiae, jaundice, hepatosplenomegaly and thrombocytopenia, and 90 % of these cases will show significant neurologic sequelae [2]. Chorioretinitis is the most common ophthalmic manifestation of congenital CMV infection, and the incidence of CMV chorioretinitis has been reported to be as high as 25 % of infants with symptomatic congenital CMV infection and ~1 % of asymptomatic infants [3]. Although no ocular abnormalities other than chorioretinitis have been reported to be associated with congenital CMV infection, we present here a case exhibiting major retinal vascular occlusions and arterio-venous anastomosis associated with CMV infection. These findings regressed dramatically after treatment with systemic gancyclovir administration.

## Case presentation

A Japanese infant boy weighing 1860 g was delivered by cesarean section at 36 weeks and three days because of fetal growth restriction (FGR) and an abnormal biophysical examination. The mother was HIV-negative but had reported a fever at four weeks of gestation, and the results of blood examination for the mother suggested a primary CMV infection because she had positive CMV-IgG and CMV-IgM titers. During the pregnancy, hepatosplenomegaly, intravertebral calcification and microcephaly were detected by ultrasonography and fetal magnetic resonance imaging (MRI). As a result, the infant was diagnosed with severe symptomatic congenital CMV infection. The presentation of the symptoms at birth was consistent with congenital CMV infection, which was confirmed by positive CMV-DNA in urine and whole blood, and he was referred to our department on his first day of life.

**Fig. 1:**
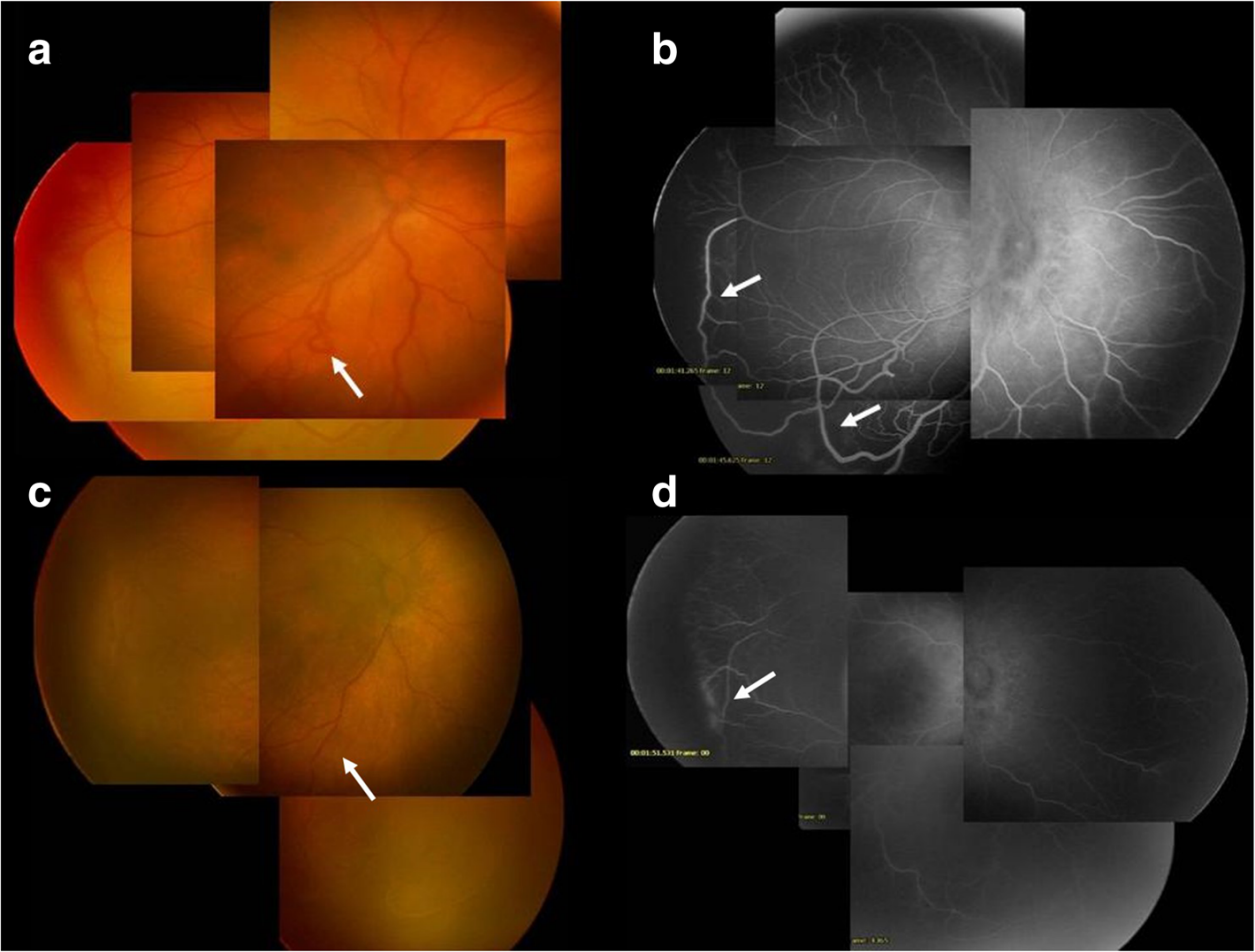
Fundus photographs and fluorescein angiography (FA) of the right eye. Ophthalmoscopic examinations revealed posterior vessel dilation in both eyes, and an arterio-venous anastomosis (arrow) in the middle of the retina in the right eye (**a**). FA revealed a filling delay and peripheral arterio-venous anastomosis (arrows). No dye leakage was found in these vessels (**b**). Typical chorioretinitis was not seen. Six days after antiviral therapy with oral valgancyclovir, the retinal vessel dilation regressed completely, and the arterio-venous anastomosis was remarkably improved (arrows) (**c**, **d**). **a** Fundus photograph on the first day of life. **b** FA of the same eye on the first day of life. **c** Fundus photograph at 11 days old (6 days of antiviral therapy). **d** FA of the same eye at 11 days old (6 days of antiviral therapy)

**Fig. 2:**
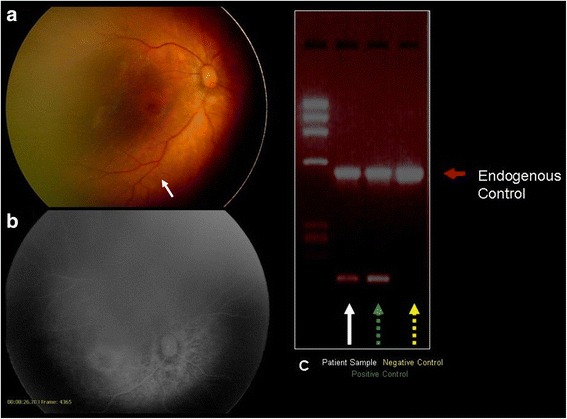
Fundus photograph and fluorescein angiography (FA) in the right eye at three months of age, and the results of the polymerase chain reaction (PCR) for the detection of CMV-DNA in the aqueous humor. The arterio-venous anastomosis initially presented in the middle of the retina had regressed (arrow); however, chorioretinal atrophy was found at the macula and around the optic disc (**a**, **b**). Result of PCR revealed intraocular CMV infection (**c**). **a** Fundus photograph at three months of age. **b** FA of the same eye at three months of age. **c** Results of the PCR for the detection of CMV-DNA (white arrow: patient sample; green dotted arrow: positive control; yellow dotted arrow: negative control; red arrow: endogenous control)

On initial consultation, a slit-lamp examination revealed no abnormalities in the cornea, iris or anterior chamber in either eye. Ophthalmoscopic examinations revealed posterior vessel dilation and a demarcation line at the temporal peripheral zone in both eyes, and an arteriovenous anastomosis in the right eye. There were no signs of chorioretinitis (Fig. 1a). On the fourth day of life, we performed fluorescein angiography (FA) for a closer examination. The results of the FA revealed a filling delay and an arterio-venous anastomosis, but no leakage from the retinal vessels was detected. On the other hand, a small avascular zone was found at the most peripheral retina (Fig. 1b). Since the findings were atypical of CMV retinitis, a sample of the aqueous humor was obtained with written informed consent, and polymerase chain reaction (PCR) was performed by SRL Inc. (Tokyo, Japan) to detect CMV-DNA in the eye. The sense and anti-sense primer sequences were TTAGTGAACCGTCAGATCGC and GCATGCATAAGAAGCCAAGG, respectively. CMV-DNA was positive in the aqueous humor (Fig. 2c). The results established an accurate diagnosis of ocular CMV infection, and the boy received systemic antiviral therapy with oral valgancyclovir (six weeks to complete treatment). Six days after the initiation of antiviral therapy with oral valgancyclovir, the vessel dilation regressed completely, and the arterio-venous anastomosis improved remarkably (Fig. 1c, d). At three months of age, we re-examined the patient using FA. The results showed that the arterio-venous anastomosis had almost completely regressed. However, chorioretinal atrophy was found at the macula and around the optic disc (Fig. 2a, b).

## Discussion

CMV is the most common congenital and perinatal viral infection and the fetus may become infected as a consequence of primary and recurrent maternal infections [2]. Five percent of all congenitally infected children have severe disease, another 5 % have mild involvement, and 90 % are born with subclinical but chronic CMV infections [4]. Long-term sequelae occur in 40–58 % of symptomatic children and in 13.5 % of asymptomatic children at birth [4]. The most characteristic neurological problems include a mild increase in cerebrospinal fluid protein, sensorineural hearing loss and chorioretinitis. Active CMV infection is confirmed by virus isolation from the urine, saliva, washings, breast milk, cervical secretions and tissues obtained by biopsy using PCR for the detection of CMV-DNA. CMV retinitis is known to occur in immunosuppressed individuals, and its incidence has increased over the last few years as a result of acquired immunodeficiency syndrome (AIDS) [5]. However, congenital CMV infection-associated retinitis may occur in patients with no systemic immune dysfunction [6]. The present patient was referred to us at 1 day of age, and he showed remarkable dilation of the retinal vessels and arterio-venous anastomoses in both eyes associated with congenital symptomatic CMV infection. Although the extent of immunosuppression was not determined, the diagnosis was made by clinical findings and cultures of blood and urine, from which the virus was isolated. These findings strongly suggested an active, systemic CMV infection. However, retinal arterio-venous anastomosis has not been previously described in conjunction with congenital CMV infection [7] except for one German article that reported aplasia of retinal vessels in a patient with congenital CMV infection [8]. As a differential diagnosis, we considered ROP due to FGR. However, it is unlikely that retinal vessel abnormalities due to ROP would promptly regress in a few days with only oral valgancyclovir. The present case showed retinal vessel abnormalities at 1 day of age after 36 weeks’ gestation. A previous report demonstrated severe retinitis and optic neuritis at 1 day of age in an infant born at 35 weeks’ gestation weighing 1180 g [9]. Interestingly, rapid and dramatic improvement of retinitis and optic neuritis was observed three days after an intravitreal injection of ganciclovir in that case; a similar rapid improvement was found in the present case. Therefore, we speculated that the fundus manifestations might be affected by the underlying state (including duration) of the ocular CMV infection although the details remain unclear. However, it is possible that our case also had mild retinitis and optic neuritis since chorioretinal atrophy at the macula and around the optic disc was subsequently found at three months of age. The mechanism describing how CMV influences retinal vascular structure is largely unknown, but previous studies showed that CMV induces proinflammatory and angiogenic cytokines in retinal pericytes [10, 11] and induces an anti-migratory and anti-angiogenic endothelial cell phenotype. These two changes could have a detrimental effect on vasculature development.

## Conclusions

We presented here the first ocular findings of congenital CMV infection. These findings might be retinal manifestations of congenital CMV infection.

## Abbreviations

AIDS, acquired immunodeficiency syndrome; CMV, cytomegalovirus; FA, fluorescein angiography; FGR, fetal growth restriction; MRI, magnetic resonance imaging; PCR, polymerase chain reaction; ROP, retinopathy of prematurity.

## References

[1] Lanzieri TM, Dollard SC, Bialek SR, Grosse SD (2014). Systematic review of the birth prevalence of congenital cytomegalovirus infection in developing countries. Int J Infect Dis.

[2] Swanson EC, Schleiss MR (2013). Congenital cytomegalovirus infection: new prospects for prevention and therapy. Pediatr Clin North Am.

[3] Istas AS, Demmler GJ, Dobbins JG, Stewart JA (1995). The National Congenital CMV Disease Registry Collaborating Group. Survelliance for CMV disease: a report from The National Congenital CMV Disease Registry. Clin Infect Dis.

[4] Dollard SC, Grosse SD, Ross DS (2007). New estimates of the prevalence of neurological and sensory sequelae and mortality associated with congenital cytomegalovirus infection. Rev Med Virol.

[5] Vancíková Z, Dvorák P (2001). Cytomegalovirus infection in immunocompetent and immunocompromised individuals--a review. Curr Drug Targets Immune Endocr Metabol Disord.

[6] Vishnevskia-Dai V, Shapira Y, Rahav G, Shimoni A, Somech R, Moisseiev J (2015). Cytomegalovirus retinitis in HIV-negative patients: a practical management approach. Ophthalmology.

[7] Ghekiere S, Allegaert K, Cossey V, Van Ranst M, Cassiman C, Casteels I (2012). Ophthalmological findings in congenital cytomegalovirus infection: when to screen, when to treat?. J Pediatr Ophthalmol Strabismus.

[8] Rochels R, Trevino E, Brand M (1983). Aplasia of retinal vessels in congenital cytomegaly (written in German). Klin Monbl Augenheilkd.

[9] Lalezary M, Recchia FM, Kim SJ (2012). Treatment of congenital cytomegalovirus retinitis with intravitreous ganciclovir. Arch Ophthalmol.

[10] Gustafsson RK, Jeffery HC, Yaiw KC, Wilhelmi V, Kostopoulou ON, Davoudi B, Rahbar A, Benard M, Renné T, Söderberg-Nauclér C, Butler LM. Direct infection of primary endothelial cells with human cytomegalovirus prevents angiogenesis and migration. J Gen Virol. 2015. doi:10.1099/jgv.0.000301. [Epub ahead of print]. 10.1099/jgv.0.000301 26416316

[11] Wilkerson I, Laban J, Mitchell JM, Sheibani N, Alcendor DJ (2015). Retinal pericytes and cytomegalovirus infectivity: implications for HCMV-induced retinopathy and congenital ocular disease. J Neuroinflammation.

